# Transcription factor PAX6 as a novel prognostic factor and putative tumour suppressor in non-small cell lung cancer

**DOI:** 10.1038/s41598-018-23417-z

**Published:** 2018-03-22

**Authors:** Yury Kiselev, Sigve Andersen, Charles Johannessen, Bjørn Fjukstad, Karina Standahl Olsen, Helge Stenvold, Samer Al-Saad, Tom Donnem, Elin Richardsen, Roy M. Bremnes, Lill-Tove Rasmussen Busund

**Affiliations:** 1 Department of Life Sciences and Health, Faculty of Health Sciences, OsloMet – Oslo Metropolitan University, Oslo, Norway; 2 Department of Pharmacy, UiT The Arctic University of Norway, Tromso, Norway; 3 Department of Clinical Medicine, UiT The Arctic University of Norway, Tromso, Norway; 4 Department of Oncology, University Hospital of North Norway, Tromso, Norway; 5 Department of Medical Biology, UiT The Arctic University of Norway, Tromso, Norway; 6 Department of Computer Science, Faculty of Science and Technology, UiT The Arctic University of Norway, Tromso, Norway; 7 Department of Community Medicine, Faculty of Health Sciences, UiT The Arctic University of Norway, Tromso, Norway; 8 Department of Clinical Pathology, University Hospital of North Norway, Tromso, Norway

## Abstract

Lung cancer is the leading cause of cancer deaths. Novel predictive biomarkers are needed to improve treatment selection and more accurate prognostication. PAX6 is a transcription factor with a proposed tumour suppressor function. Immunohistochemical staining was performed on tissue microarrays from 335 non-small cell lung cancer (NSCLC) patients for PAX6. Multivariate analyses of clinico-pathological variables and disease-specific survival (DSS) was carried out, and phenotypic changes of two NSCLC cell lines with knockdown of PAX6 were characterized. While PAX6 expression was only associated with a trend of better disease-specific survival (DSS) (p = 0.10), the pN+ subgroup (N = 103) showed significant correlation between high PAX6 expression and longer DSS (p = 0.022). Median survival for pN + patients with high PAX6 expression was 127.4 months, versus 22.9 months for patients with low PAX6 expression. In NCI-H661 cells, knockdown of PAX6 strongly activated serum-stimulated migration. In NCI-H460 cells, PAX6 knockdown activated anchorage-independent growth. We did not observe any significant effect of PAX6 on proliferation in either of cell lines. Our findings strongly support the proposition of PAX6 as a valid and positive prognostic marker in NSCLC in node-positive patients. There is a need for further studies, which should provide mechanistical explanation for the role of PAX6 in NSCLC.

## Introduction

Despite extensive efforts of research and arrival of novel therapies, lung cancer remains leading cause of cancer death globally^1^ and in the United States^2^. It is the second leading cause of death from any cause after heart disease in the United States^3^. The 5-year relative survival rate for lung cancer is only about 17%^4^, in particular due to a high proportion of patients with regional or advanced disease at the time of diagnosis^5^. Treatment of metastatic non-small cell lung cancer (NSCLC) has limited effect because of either intrinsic or acquired resistance to chemotherapy^6^.

In 2002 it was stated that a plateau was reached for treatment efficacy in NSCLC, which could not be improved further with conventional chemotherapeutic drugs^7^. Yet, some progress has been achieved since then^8^, mainly by the introduction of new targeted drugs and patient selection based on histological subtypes and driver mutations, which define the course of disease and predict drug efficacy^9,10^. Novel predictive biomarkers for NSCLC are needed to allow improved treatment selection and more accurate prognosis^11^.

Transcription factors in general are tightly connected both to normal development and to cancer^12^. PAX6 is a highly evolutionary conserved transcription factor, belonging to the paired box family. It plays an important role in the development of the central nervous system, eyes, nose and endocrine pancreas^13^. In 2003, PAX6 mRNA expression was first described in a wide range of cancer cell lines^14^. However, only a limited number of studies have investigated PAX6 expression in human malignancies, its biological role in cancer and possible effects on survival. Several publications link methylation of the PAX6 gene promoter with patients’ prognosis, and discuss its relevance as a biomarker in NSCLC^15–17^, as well as bladder cancer^18^, gastric cancer^19^, oesophageal cancer^20^, and breast cancer^21^. Only two groups have evaluated the prognostic impact of PAX6 in cancers using gene expression data obtained from clinical samples. In gliomas, higher levels of PAX6 mRNA were associated with better survival^22,23^. Opposite association was found in a small study of neuroendocrine^24^ and breast tumors^25^. Molecular biology evidence for PAX6 acting as a tumor suppressor exists mainly for glioblastoma^26–28^, retinoblastoma^29^ and prostate cancer^30^ cells lines. In contrast, a tumour promoting effect was demonstrated in retinoblastoma^31^, colon adenocarcinoma^32^ and breast cancer^33^ cell lines. In view of the cancer cell lines data and more abundant knowledge of the role of PAX6 in normal development^34^, PAX6’s exact functions appear to depend on tissue-, molecular- and spatio-temporal contexts. So far, prognostic significance of PAX6 protein expression has been investigated only in one study of breast cancer^25^. Herein, we have investigated the PAX6 protein expression and evaluated its prognostic impact in a large NSCLC cohort.

## Results

### Patient characteristics

The patients’ demographic, clinical and histopathological data are presented in Table 1. The median follow-up time of survivors was 86 months (range 48–216). The median patient age was 67 (range 28–85), 76% were male, 95% had performance status 0–1 and 95% were present or previous smokers. The NSCLC tumours comprised 191 squamous cell carcinomas (SCC), 14 adenocarcinomas (AC), 9 adenocarcinomas *in-situ* (ACIS) and 31 large-cell carcinomas (LCC).

**Table 1:** Prognostic clinicopathologic variables as predictors of disease-specific survival in 335 NSCLC-patients.

Characteristics	Patients N, (% of total)	Median survival (months)	5-year survival (%)	P
Age				0.42
≤65 years	156 (47)	98	56	
>65 years	179 (53)	NR	60	
Sex				0.22
Female	82 (24)	190	64	
Male	253 (76)	98	56	
Smoking status				0.26
Never	15 (5)	19	43	
Previous	105 (31)	84	55	
Present	215 (64)	NR	60	
Performance status				**0**.**016**
0	197 (59)	NR	63	
1	120 (36)	64	52	
2	18 (5)	25	33	
Weight loss				0.76
<10%	303 (90)	190	58	
>10%	32 (10)	98	57	
Histology				**0**.**028**
Squamous cell carcinoma	191 (57)	NR	66	
Adenocarcinoma	113 (34)	54	46	
Large cell carcinoma	31 (9)	98	56	
Differentiation				** <0**.**001**
Poor	138 (41)	47	47	
Moderate	144 (43)	190	65	
Well	53 (16)	NR	68	
Surgical procedure				**0**.**007**
Wedge + Lobectomy	243 (73)	190	62	
Pneumectomy	92 (27)	37	47	
pStage				** <0**.**001**
pI	157 (47)	NR	72	
pII	136 (41)	62	51	
pIIIA	42 (12)	17	24	
pT-status				** <0**.**001**
1	85 (25)	190	75	
2	188 (56)	84	57	
3	62 (19)	25	37	
pN-status				** <0**.**001**
0	232 (69)	NR	67	
1	76 (23)	35	43	
2	27 (8)	18	18	
Surgical margins				0.37
Free	307 (92)	190	59	
Not free	28 (8)	47	48	
Vascular infiltration				** <0**.**001**
No	284 (85)	190	62	
Yes	51 (15)	27	33	
Pax6 expression	335 (100)			0.10
High	94 (28)	NR	67	
Low	241 (72)	75	55	
Pax6 expression in AC	113 (34)			0.32
High	22 (7)	NR	57	
Low	91 (27)	47	44	
Pax6 expression in SCC	191 (57)			0.52
High	60 (18)	NR	73	
Low	131 (39)	NR	63	
Pax6 expression in pN+	103 (31)			**0**.**022**
High	33 (10)	127	59	
Low	70 (21)	23	26	

AC – adenocarcinoma, NR – not reached, SCC – squamous cell carcinoma.

### Validation of the primary antibody

**Figure 1:**
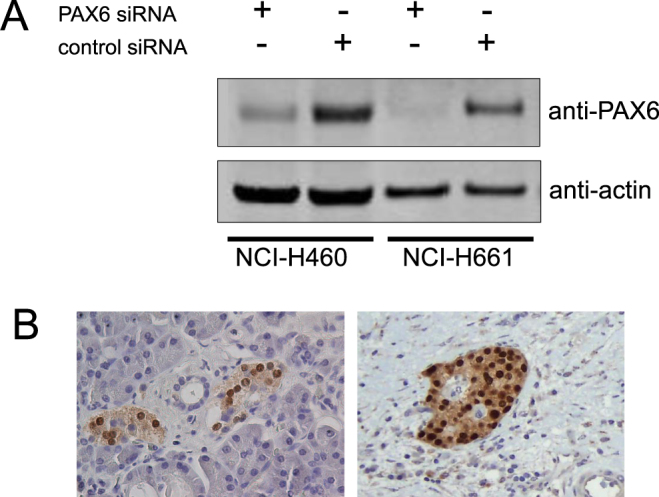
Validation of PAX6 antibody. (**A**) Two NSCLC cell lines were transfected with siRNA against human PAX6 or control scrambled siRNA. Cell lysates were tested for PAX6 using Western blotting. (**B**) FFPE sections of pancreas with Langerhans islands stained for PAX6.

In order to validate specificity and sensitivity of the chosen primary antibody, we employed Western blotting and immunohistochemistry (IHC). Two human NSCLC cell lines with endogenous expression of PAX6 were transfected with either PAX6 siRNA or scrambled control siRNA, incubated for 48 hours, lysed and analysed by Western blotting (Fig. 1A; see Fig. S1 for uncropped image). A single band of appropriate molecular weight (approximately 48 kD) was observed for both cell lines, and significant reduction of the band intensity was noted in samples treated with anti-PAX6 siRNA. Equal loading was controlled by staining for actin.

To further validate the anti-PAX6 antibody for use on human FFPE tissue sections, we chose a fragment of pancreatic tissue with Langerhans islets, which are known to contain PAX6-positive cells^35,36^ (Fig. 1B), as positive control. Indeed, we observed clear strong nuclear staining with much weaker staining in the cytoplasm. No staining was seen in the exocrine part of the pancreas.

### Expression of PAX6 in NSCLC tissues

**Figure 2:**
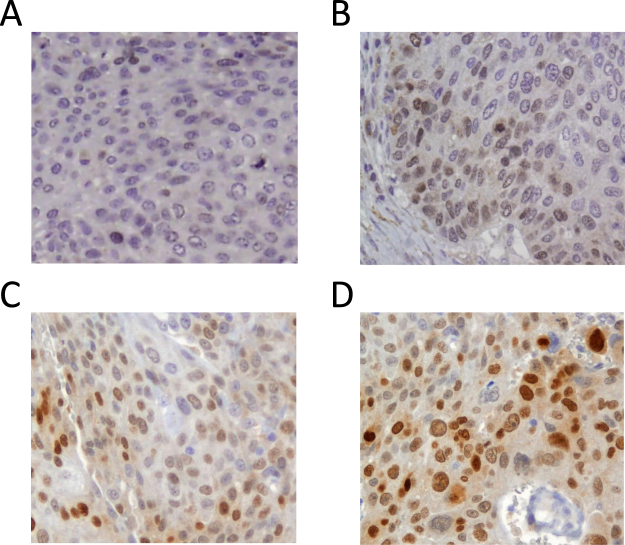
Immunohistochemical staining for PAX6 in NSCLC. NSCLC tissue with negative (**A**), weak (**B**), average (**C**) and strong (**D**) staining for PAX6.

We observed nuclear staining for PAX6, often accompanied by moderate cytoplasmic staining (Fig. 2). It was detectable exclusively in NSCLC tumour cells, and not in the surrounding tumour stroma, or adjacent normal lung tissue. Pattern of positive cells varied across the TMA cores from patchy to diffuse. However, we did observe a tendency of higher staining intensity in the tumour cells located in proximity to stroma or next to the invasion front. A weak cytoplasmic (but not nuclear) staining was sometimes seen in macrophages, which we considered an artefact. Neither nuclear nor cytoplasmic staining were observed in adjacent normal lung tissue.

### Expression correlations

We found no correlations between PAX6 and clinicopathological variables. There were a lot of low grade, but significant correlations, when the PAX6 expression was compared with tumour cell expression of molecular markers previously published by our group. Only correlations with a *r*-value > 0,2 are presented; IGFBP2 (r = 0.258, p < 0.001), BAD (r = −0.20, p < 0.001), HER1 (r = 0.247, p < 0.001), PTEN (r = 0.241, p < 0.001), sAKT (the activated isoform at ser474) (r = 0.292, p < 0.001), trombospondin (r = 0.288, p < 0.001), MMP7 (r = 0.272, p < 0.001), TIE2 (r = 0.208, p < 0.001), DLL4 (r = 0.233, p < 0.001) and PDGFA (r = −0.278, p < 0.001).

### Survival analysis

**Figure 3:**
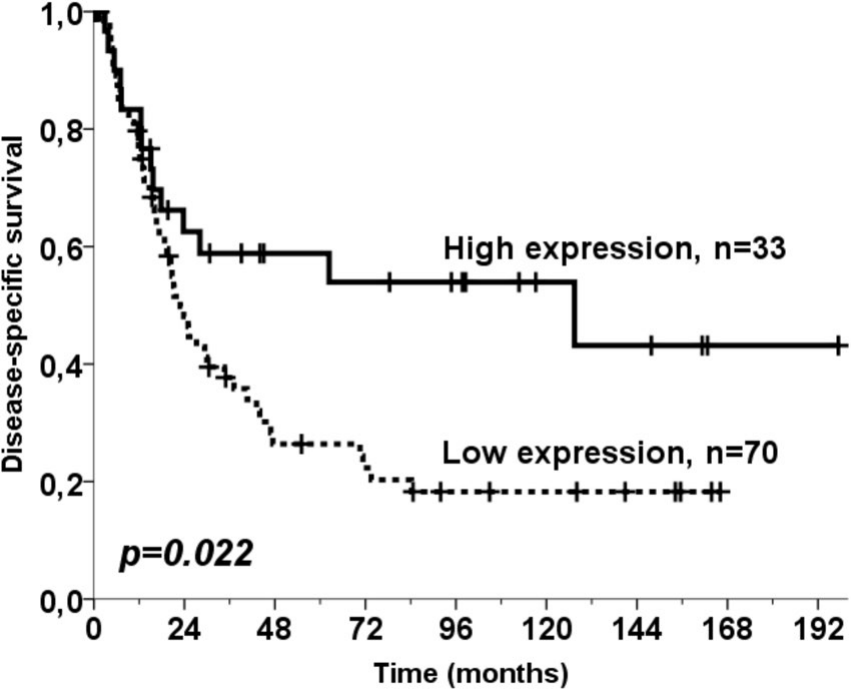
Disease-specific survival according to PAX6 expression. Disease-specific Kaplan-Meier survival curves according to PAX6 expression in 103 resected NSCLC patients with regional nodal metastasis (pN+). The P-value is according to the log-rank test.

High PAX6 expression was associated with a trend for better DSS (p = 0.10). When stratifying for relevant clinical subgroups, the pN+ subgroup (N = 103) showed a significant association between high PAX6 expression and a longer DSS (p = 0.022, Fig. 3). Median survival for pN+ patients with high PAX6 expression was 127.4 months whereas the same value for patients with low PAX6 expression was only 22.9 months. Five-year survivals for the same groups were 59% and 26%, respectively.

In the pN+ subgroup, no other clinical or pathological variables had any prognostic significant impact in univariate analysis. Therefore, the multivariate model only included PAX6 expression which gave an unadjusted hazard ratio of 1.99 for the low expression versus high expression subgroup (p = 0.024).

To assess PAX6 impact on prognosis in the pN+ category in a model with renowned clinicopathological factors, we also introduced WHO PS and pTstage in the model. But these were knocked out in the backward conditional model. In the “enter” model which does not remove factors, the impact of PAX6 was even stronger (P = 0.022 and HR = 2.08) than unadjusted.

### Effect of PAX6 on NSCLC cell migration, proliferation and anchorage-independent growth

**Figure 4:**
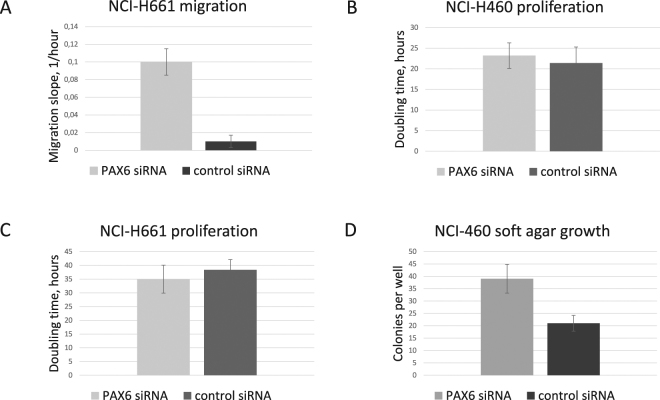
Phenotypic effects of PAX6 knockdown on NSCLC cell lines. (**A**) Migration of NCI-661 cells across porous membrane. (**B**) Proliferative activity of NCI-H460 and (**C**) NCI-661 cells. (**D**) soft agar colony formation assay in NCI-H460 cells. PAX6 knockdown efficiency is demonstrated in Fig. 5.

**Figure 5:**
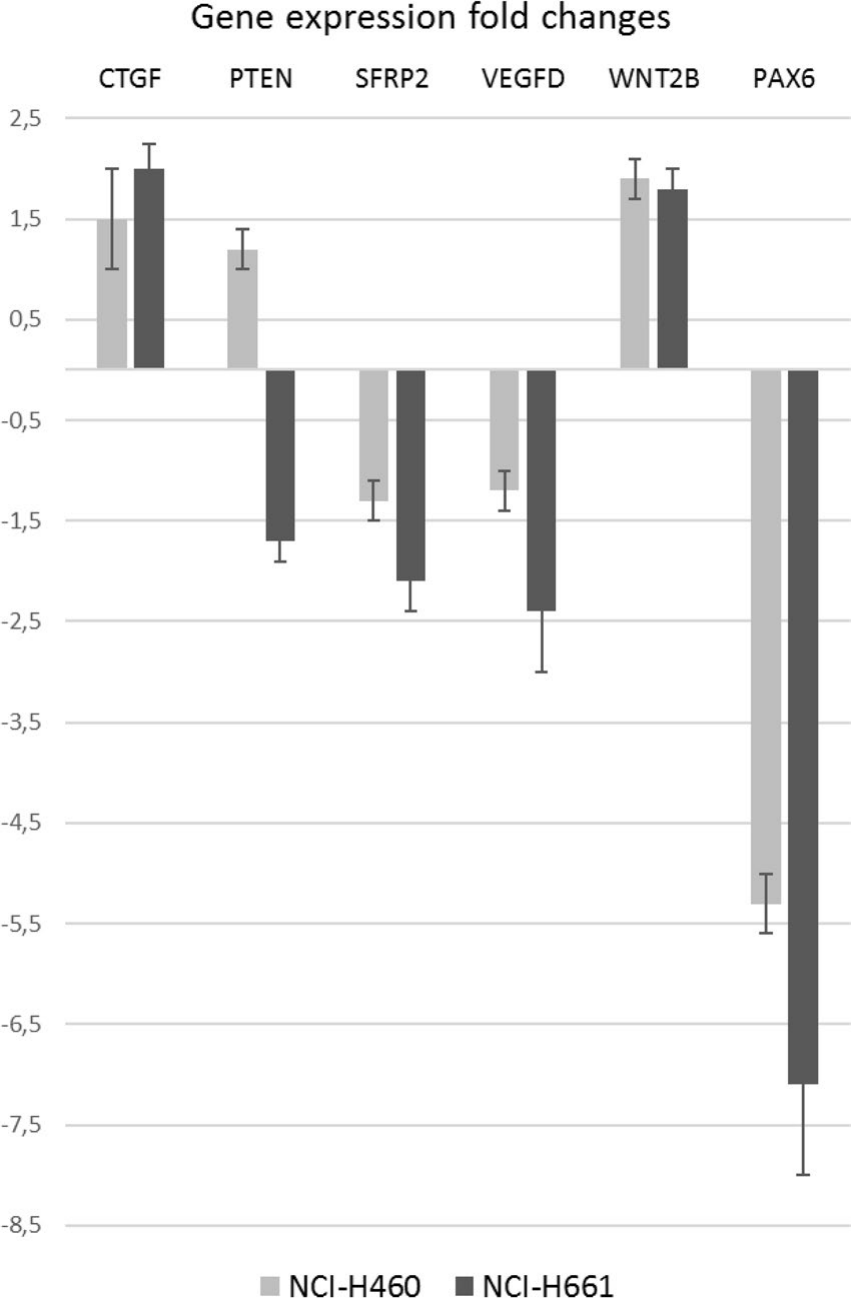
Putative downstream target genes of PAX6 in NSCLC. PAX6 was transiently knocked down in two NSCLC cell lines. Expression of the target genes was measured by real-time qPCR. The amount of target gene was normalized to the average expression of the two human reference genes GUSB and TFRC, and is shown relative to scrambled siRNA-treated control.

In order to uncover possible biological effects of PAX6 in NSCLC cells, we evaluated effects of transient knockdown of PAX6 on cell migration, proliferation and anchorage-independent growth. To study proliferation and migration in real time, we employed the xCELLigence platform (Roche). This is a microelectric assay based on changing impedance of bottom electrodes in presence of the cells. In NCI-H661 cells, knockdown of PAX6 strongly activated migration capability, measured as the serum-stimulated migration on xCELLigence platform (Fig. 4A) and supported by the wound healing assay in the Incucyte instrument (Supplementary Fig. 3). NCI-H460 cells did not exhibit ability to migrate across the membrane after transfection with either PAX6 siRNA or control siRNA. We did not observe any significant effect of PAX6 on proliferation rate of either cell lines (NCI-H460 and NCI-H661) when measured by xCELLigence platform (Fig. 4B,C). NCI-H460 cells subjected to PAX6 knockdown exhibited profoundly more active anchorage-independent growth in soft agar, compared to controls (Fig. 4D). Neither treated nor control NCI-H661 cells were capable of making colonies in soft agar assay. Efficiency of knockdown was controlled using RT-PCR: fold change for PAX6 in NCI-H460 cells was −5,3 ± 0,3, and in NCI-H661 −7,1 ± 0,9 (Fig. 5).

### Effect of PAX6 on expression of selected NSCLC-relevant genes

In order to screen for possible molecular targets of PAX6 in NSCLC, we used real-time qPCR to measure changes of mRNA expression of the panel of 25 genes upon PAX6 knockdown. Selection of genes included in the panel was based on previous reports on predictive biomarkers published by our group^37–43^, as well as studies of downstream targets of PAX6 performed by other groups^44–48^. We chose the threshold fold change of 1,5 (or −1,5) as minimum sufficient to consider a gene to be affected by PAX6 knockdown. Noteworthy, in the NCI-H661 cell line, PAX6 knockdown caused moderate but reproducible upregulation of CTGF, and downregulation of PTEN, VEGFD and SFRP2. While in the NCI-H460 upregulation of WNT2B was of note (Fig. 5).

### Assessment of the correlation between PAX6 and target genes

To confirm the association between PAX6 expression and PAX6 target genes, we assessed correlations in a compendium of 18 000 gene expression data sets available from GEO using the ChIPXpress R package. Results show overall low scores, meaning high correlations between expression levels of PAX6 and the target genes (Table 2).

**Table 2:** Prediction and ranking of functional PAX6 target genes based on publicly available GEO datasets. Genes are ranked according to the ChIPXpress score, where a small score corresponds to a highly ranked gene.

Rank	Gene Symbol	Entrez GeneID	ChIPXpress score
1	WNT2B	7482	1.2
2	SFRP2	6423	1.9
3	CTGF	1490	2.9
4	PTEN	5728	4.0
NA	VEGFD (FIGF)	2277	NA

## Discussion

To our knowledge, we are the first group to report PAX6 protein expression in NSCLC. In the total NSCLC cohort we observed a trend towards longer survival in patients with high PAX6 expression. Among patients with nodal metastases, those with high PAX6 expression showed significantly increased survival.

Previous reports describing PAX6 expression in human malignancies dealt with tumours of brain and pancreas, which are the organs with tight developmental links to PAX6. Expression of PAX6 in lung cancer is a somewhat surprising finding, yet it is expected of malignant tumours to show gross aberrations in expression profiles compared to the corresponding normal tissues. We have previously experienced difficulties with finding a commercially available specific antibody against PAX6, which could be used for both Western blotting and staining of FFPE tissue sections. Several of the tested antibodies (marketed by manufacturers as specific for human PAX6) did produce bands of nearly correct size on Western blots, and did stain various cell types using IHC, but were shown to be non-specific in our validation tests. The antibody used in this study performed very well on a Western blot with cells stably overexpressing PAX6^44^ and siRNA-treated cells with endogenous expression of PAX6 (Fig. 1A). In FFPE pancreatic tissue sections it stained exclusively endocrine islets (Fig. 1B).

Statistical analyses of our TMA data revealed a non-significant trend for better DSS in patients with higher expression of PAX6. However, significantly improved survival was found among node-positive patients (P = 0.022). There is a lack of validated biomarkers for node-positive disease, which represents a substantial proportion of patients with NSCLC. Involvement of regional lymph nodes is a major event which decreases chances for survival and dictates a need for adjuvant or neoadjuvant chemotherapy^10^. Therefore, emergence of PAX6 as a beneficial prognostic marker for node-positive patients is important. Further studies should dissect the molecular mechanisms explaining the tumour-suppressive action of PAX6, as well as evaluate whether its expression may predict response to anticancer therapy such as platinum-based therapy and novel targeted therapies.

In an attempt to explore the biological effect of PAX6 in NSCLC, we analysed changes in proliferation, migration and anchorage-independent growth after cells were transfected with siRNA against PAX6. Several independent experiments did not provide significant evidence of shifts in proliferative activity of NSCLC cells upon transient knockdown of PAX6. However, a prominent effect was seen for migration capacity of NCI-H661 cells. Cells transfected with PAX6 siRNA were able to migrate actively through the porous membrane in CIM-plates along the serum gradient. Compared to these, control cells demonstrated very little if any migration. Similar results were obtained from the scratch assays analysed by the camera-based real-time cell monitoring instrument Incucyte (not shown). Inhibition of cancer cell migration by PAX6 fits well with the proposed tumour-suppressor role and with our survival data in pN+ patients. Although PAX6 is known to be a crucial regulator of cell migration during development of the eye and the brain^34^, as well as influencing cell migration when exogenously expressed in normal murine fibroblasts^44^, there are to our knowledge no publications on the effect of PAX6 on migration of cancer cells.

Overcoming anoikis and becoming capable of anchorage-independent growth is one of the core events in the malignant transformation of cells, as well as in establishing new tumour foci beyond the primary site^49^. In our study, PAX6-deficient NSCLC cells were able to grow much more actively in soft agar, than control cells. It is possible that in patients with node-positive disease, PAX6 decelerates further dissemination of the tumour and thus improves survival. Again, this is in line with our concept that PAX6 acts as a tumour suppressor in NSCLC. Others have shown that PAX6 increases glioma cell susceptibility to detachment an oxidative stress^28^. Zhao *et al*. indicated that A549 and H1299 NSCLC cell lines with stable knockdown of PAX6 had a lower rate of colony formation in soft agar compared to controls^50^. We had tested the mentioned cell lines (along with 5 other lines) for endogenous expression of PAX6 protein prior to starting the cell experiments for our study. We have observed that A549 and H1299 cells had very low levels of PAX6, when compared to the two cell lines which we chose to continue with (NCI-H460 and NCI-H661). Zhao *et al*.^50^ used a stable knockdown of PAX6 instead of a transient one, which potentially could lead to adaptations of cells to long-term deficit of PAX6 and alteration of related regulatory networks. This might explain the discrepancy between our results with respect to anchorage-independent growth. Luo *et al*. report that PAX6 activates A529 cell proliferation and invasion^51^, yet one may argue that overexpressing PAX6 in a cell line with very low endogenous level of PAX6 might be less optimal approach than inducing knockdown of PAX6 in cells with high endogenous levels of PAX6.

The observed changes in expression of NSCLC-relevant genes may contribute to understanding of the molecular mechanisms behind PAX6’s role in NSCLC. PAX6 upregulates a known tumor suppressor PTEN, as well as SFRP2 - an inhibitor of the WNT pathway. The genes inhibited by PAX6 (WNT2B and CTGF) have been previously shown to have pro-malignant roles in NSCLC^52–54^. SFRP2 and WNT2B belong to the WNT pathway, activation of which is important in lung cancer biology^55^. Our data suggest that PAX6 may inhibit WNT pathway through upregulation of its suppressor (SFRP2) and downregulation of its effector (WNT2B).

For in silico validation of PAX6 target genes, the ChIPXpress approach provides improved target gene prediction compared to experimental information or database information alone^56^. We identified high correlations between PAX6 and the predicted target genes, which lends strength to our experimental findings. However, ChIPXpress relies on the assumption that transcription factor binding induces gene expression changes, which is not always the case. For example, genes may be regulated by numerous transcription factors collectively and variation in on transcription factor may not induce expression change. Further, ChIPXpress may not be able to properly rank genes that are lowly expressed, or genes that are regulated only in specific tissues or conditions. Lastly, ChIPXpress does not differentiate between positive or negative correlation, but rather indicates all regulated genes.

The tumour suppressor role of PAX6 in NSCLC, which we propose in this paper, is in line with a number of methylation studies, which indicated that PAX6 promoter methylation is common in several cancers^18^ -^21^, including NSCLC^15–17^, and is associated with disease recurrence^18^ and poor survival^17,19^.

## Conclusion

We believe that our findings support the proposition of PAX6 as a valid and positive prognostic marker in NSCLC in node-positive patients. There is a need for further studies, which should provide further mechanistical explanation for the role of PAX6 in NSCLC, in particular regarding target genes of PAX6 in this tumour group.

## Materials and Methods

All methods were performed in accordance with the relevant guidelines and regulations.

### Patients and tissues

Retrospectively, we identified 371 patients who were surgically resected with pathological stage I to IIIA NSCLC at the University Hospital of North Norway and Nordland Central Hospital between 1990 and 2004. Primary tumour tissue was collected from the archives of the two respective pathological departments. After exclusion of 36 patients due to inadequate paraffin-embedded formalin-fixed tissue blocks (n = 13), other malignancy within 5 years prior to diagnosis (n = 13) or radiotherapy or chemotherapy prior to surgery (n = 10), we were left with 335 eligible patients with complete demographic and clinicopathological data. The histopathological data were revised according to the 7th edition of UICC TNM classification of lung cancer and the newly revised classification for NSCLC adenocarcinoma^57,58^. A total of 103 patients had metastases to regional lymph nodes (pN+). Adjuvant chemotherapy was not yet introduced in Norway during this period (1990–2004). The last disease-specific survival (DSS) update was done in November 2011.

The Regional Committee for Medical and Health Research Ethics (2009/1393), the Data Protection Official for Research (NSD), and the Norwegian National Data Inspection Board approved this study. All patients were anonymized with each trial number. These numbers were initially linked to identity for only one purpose prior to collect clinical information. The Norwegian Social Science Data Service and the University Hospital’s Data Protection Office accepted this solution (2009/1393). Written consent from the patients was considered, but as this was a retrospective study where most of the material was more than 10 years old and most of the patients deceased, it was considered not needed. All data was analysed anonymously.

### Microarray construction

Duplicate 0.6 mm core biopsies from the most representative areas of tumour cells (neoplastic epithelial cells) and tumour-surrounding stroma were collected from each surgical specimen using a tissue-arraying instrument (Beecher Instruments, Silver Springs, MD). Normal lung tissue localized distant from the primary tumour as well as lung tissue samples from 20 normal lungs from patients without any history of malignancy were also sampled. Eight tissue microarray blocks (TMAs) were constructed to include all the cores. The detailed methodology has been reported previously^43,59^.

### Immunohistochemistry (IHC)

Sections were deparaffinised with xylene and rehydrated with ethanol. Antigen retrieval was performed by placing the specimen in 0.01 mol/l citrate buffer at pH 6.0 and exposed to two repeated microwave heatings of 10 minutes at 450 W. The DAKO EnVision + System-HRP (DAB) kit was used as endogen peroxidase blocking. Primary antibody against PAX6 (AB2237, Millipore) was incubated overnight at 4 °C. The DAKO EnVision + System-HRP (DAB) kit was used to visualize the antigen-antibody complexes. This yielded a brown reaction product at the site of the target antigen. As negative staining controls, the primary antibodies were replaced with the primary antibody diluent. All TMA, including the negative controls, were stained in one single experiment. Finally, TMA slides were counterstained with haematoxylin to visualize the nuclei.

### Scoring of immunohistochemistry

Viable parts of each anonymized core were scored independently and semiquantitatively by one pathologist and one oncologist, who was trained to score immunohistochemically stained sections (S.A.S and H.S) by light microscopy. When assessing a variable in a given core, the scorers were blinded to the outcome and score of the other observer. Only the neoplastic cell compartment (tumour epithelial cells) was scored in this study as there was no expression detected in the tumour-surrounding stroma. The dominant staining intensity in tumour was scored as: 0 = negative, 1 = weak, 2 = intermediate or 3 = strong. We observed nuclear staining of varying intensity for PAX6, as well as some degree of cytoplasmic staining in cells with nuclear positivity. Nuclear and cytoplasmic staining as well as the density of tumour cells positive for PAX6 were scored separately.

A mean score was calculated for the two tumour cell cores for each individual. In tumour, high expression was defined as intensity of nuclear expression ≥1. Similar scoring methods have been used in our previous IHC-scoring studies^43,60,61^ and by others^62^. Optimal statistical cut-off levels for high and low expression were used.

### Cell cultures

Of seven NSCLC cell lines available at our lab only two had significant levels of endogenous PAX6 expression (NCI-H460 and NCI-H661), and those were chosen for experiments. Human large cell lung carcinoma cell lines NCI-H661 (ATCC HTB-183) and NCI-H460 (ATCC HTP-177) were grown in RPMI-1640 (Sigma-Aldrich) with 10% FCS. Media for all cell lines contained 1% of mixed 100 U/ml penicillin and 100 mg/ml streptomycin (cat#P0781, Sigma-Aldrich).

### Western blotting

Cells were lysed directly in the NuPAGE® LDS Sample Buffer (NP0007, Life Technologies) with DTT. Samples were run on NuPAGE Novex 4–12% Bis-Tris gels (NP0322, Life Technologies), and blotted onto the Odyssey nitrocellulose membrane (LI-COR Biosciences, Lincoln, NE). Membranes were blocked for 1 hour in room temperature using Odyssey blocking buffer (LI-COR Biosciences). Primary and secondary antibodies were diluted in the blocking buffer, and membranes were incubated at room temperature for at least 1 hour. Washing was done after each antibody incubation 5 times for 5 minutes in TBS containing 0.1% Tween-20. Membranes were incubated with IRDye Coupled secondary antibodies, and images were acquired with the Odyssey Sa Infrared Imaging System (LI-COR Biosciences).

Antibodies used were rabbit polyclonal anti-Pax6 antibody 1:1200 (AB2237, Merck Millipore), rabbit anti-Actin 1:2000 (A2066, Sigma-Aldrich), anti-rabbit 800 CW 1:10 000 (#926-32214 and #926-32213, LI-COR Biosciences). Molecular weight markers used were SeeBlue Plus2 Prestained Standard (Invitrogen) and MagicMark XP Western Protein Standard (Invitrogen).

### RNA interference

Cells were transfected in 6-well plates with human PAX6 siRNA (#114168 Silencer Select PAX6 siRNA, Ambion) using Lipofectamine 2000 (#11668–027, Invitrogen). A scrambled negative control (SCR) siRNA was included in all experiments (Silencer Negative Control #2 siRNA, Ambion). Cells were harvested 48 hours after siRNA transfection. Transfection efficiency was routinely controlled using the BLOCK-iT fluorescent oligoes (#2013, Invitrogen) with typical proportion of transfected cells 85–100%. Knockdown of PAX6 was verified by Western blot and RT-qPCR.

### RNA extraction and RT-qPCR

Total RNA was extracted using the RNeasy Plus kit (#74134, Qiagen, Hilden, Germany). Reverse transcription of total RNA was performed with Superscript III Reverse Transcriptase kit (#18080–044, Invitrogen), using 150 ng random hexamer primers (Fermentas International Inc., Canada). dNTP mix was purchased from Promega (Madison, WI). We used 500 ng total RNA per 20 µl cDNA reaction. Primer pairs were designed using Primer 3 software (Whitehead Institute, Cambridge, MA) and synthesized by Invitrogen or Sigma, or purchased directly from Qiagen (supplementary Figure 2). For quantification of mRNA expression levels, a Stratagene MX3000P instrument (Stratagene, La Jolla, CA) was used. cDNA was amplified for 40 cycles in a 25 µl SYBR green PCR mix (Brilliant II SYBR Green QPCR master mix, Stratagene) containing 200 nM of each primer. Cycling conditions were as follows: 95 °C for 10 min followed by 40 cycles at 95 °C for 30 sec, 60 °C for 1 min and 72 °C for 30 sec. Duplicate PCR analyses were performed on each cDNA sample. The absence of genomic DNA and contaminations were confirmed by the inclusion of no reverse transcriptase (No RT) controls and no template controls (NTCs) respectively. The relative amount of target gene normalized to the average expression of the two human reference genes *GUSB* and *TFRC* was determined using the ΔΔCT-method^63^.

### Assessment of the correlation between PAX6 and target genes

Correlation of gene expression levels between PAX6 and candidate target genes was assessed using ChIPXpress^56^. ChIPXpress is an R/Bioconductor package for prediction of functional transcription factor target genes through the combination of experimental results and large amounts of publicly available gene expression data from databases such as Gene Expression Omnibus (GEO, www.ncbi.nlm.nih.gov/geo/). The method is based on the observation that in spite of the inherent heterogeneity of GEO datasets, the global correlation between a transcription factor and its possible target genes provides valuable information for functional ranking of the target genes. Briefly, a compendium of more than 18 000 gene expression samples from Affymetrix Human U133 Plus 2.0 microarrays have been normalized and standardized, and is provided as part of the ChIPXpress package. ChIPXpress combines the ranked list of target genes from the researcher’s own experiments with a ranking of target genes based on the GEO compendium. The output is a linear combination of the ranks, hence, a small score corresponds to a highly ranked gene. For more details, please refer to Wu *et al*.^56^.

### Proliferation and migration assays

Assays were performed on the microelectric impedance-based xCELLigence platform (Roche) using E-plates (Roche, cat# 05469830001) for proliferation, and CIM-plates 16 (Roche, cat# 05665817001) for migration as described previously^44^. Optimal cell numbers per well (5000 NCI-H460 and 3000 NCI-H661 for proliferation and 20000 NCI-H661 for migration) were determined in initial titration experiments. At least 4 independent proliferation experiments, and 3 independent migration experiments were performed.

### Colony formation in soft agar

Colony formation in soft agar was performed by growing 1800 H460 cells in the upper layer of a two layer agar (500 μl 0.5% top agar and 500 μl 0.8% base agar) per well in a 12-well dish. Prior to growing in soft agar, cells were transfected with PAX6 siRNA and negative control (SCR) siRNA, respectively, for 6 hours in a 6-well dish. After 3 weeks cells were fixated with 500 μl 10% methanol + 10% acetic acid per well for 20 minutes. Cell colonies were stained with 500 μl 0.01% crystal violet per well for 18 hours at 4 °C before counting.

### Statistical methods

The statistical analyses were done using the SPSS 21 package (Chicago, IL). The χ^2^ test and Fishers exact tests were used to examine the associations between molecular marker expressions and the clinicopathological markers. *r*-values are the Spearman’s rank correlation coefficient. Plots of the DSS, according to marker expressions, were drawn using the Kaplan-Meier method, and the statistical significance between survival curves was assessed by the log rank test. The chosen endpoint, disease-specific survival (DSS), was calculated from the time of surgery to the time of lung cancer death.

In the multivariate analysis, statistically significant variables from the univariate analyses (P < 0.05) for pN+ patients were entered in a backward stepwise Cox regression analysis with a probability for stepwise entry and removal at 0.05 and 0.10, respectively. A P < 0.05 was considered statistically significant for all analyses.

## Electronic supplementary material

Supplementary figures

Dataset 1
