# Supplementary material for: Transcription factor PAX6 as a novel prognostic factor and putative tumour suppressor in non-small cell lung cancer
Source: Sci Rep. 2018 Mar 22;8:5059. doi: 10.1038/s41598-018-23417-z (PMC5864921; doi:10.1038/s41598-018-23417-z)

Transcription factor PAX6 as a novel prognostic factor and putative tumour suppressor in non-small cell lung cancer.

Yury Kiselev MD, PhD, Sigve Andersen MD, PhD, Charles Johannessen MSc, Bjørn Fjukstad MSc, Karina Standahl Olsen PhD, Helge Stenvold MD, PhD, Samer Al-Saad MD, PhD, Tom Donnem, MD, PhD, Elin Richardsen, MD, PhD, Roy M. Bremnes, MD, PhD, Lill-Tove Busund, MD, PhD.

Supplementary figure 1. Uncropped image of anti-PAX6 Western blot. Image was taken using an Odyssey system so that it was possible to discriminate between PAX6 (≈48kD) and actin (40 kD) bands using two different colours. This figure is monochrome, corresponding bands are indicated with arrows.

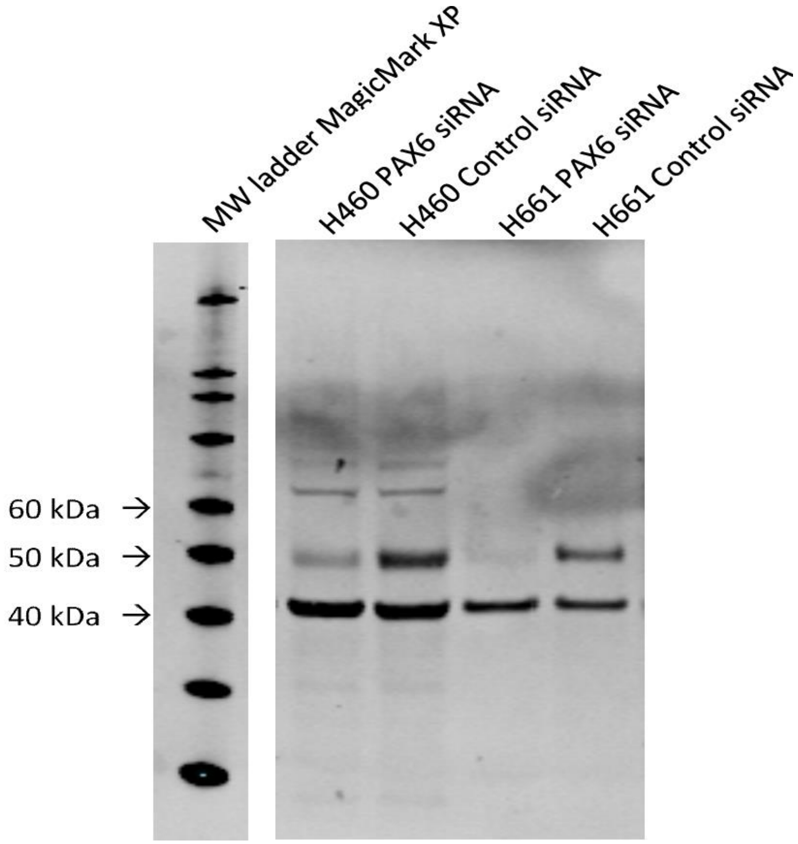

Supplementary figure 2. Sequences of primers used in real-time qPCR.

Gene	Forward primer (5' → 3')	Reverse primer (5' → 3')
**CTGF**	CAGCATGGACGTTCGTCTG	AACCACGGTTTGGTCCTTGG
**PAX6**	CAACTCCATCAGTTCCAACG	TGGATAATGGGTTCTCTCAAACTCT
**PTEN**	TGTTCAGTGGCGGAACTTGCAAT	CACAGGTAACGGCTGAGGGAACT
**WNT2b-1**	ATGTTGGATGGCCTTGGAGTGGT	ACGCTGACTGTGTAGGTATGCCA
**WNT2b-2**	TGTCCGTTTTGCCAAGGCCTTC	ACTCACGCCATGGCACTTACACT
**SFRP2**	ACATGCTTGAGTGCGACCGTTT	GCAGGCTTCACATACCTTTGGAG
**VEGFD**	ATGCAGGCTGAGGCTCAAAAGTT	GGCTGCACTGAGTTCTTTGCCAT

Supplementary figure 3. Representative pictures of wound healing assay performed on NCI-H661 cells with and without PAX6 knockdown in the Incucyte instrument.

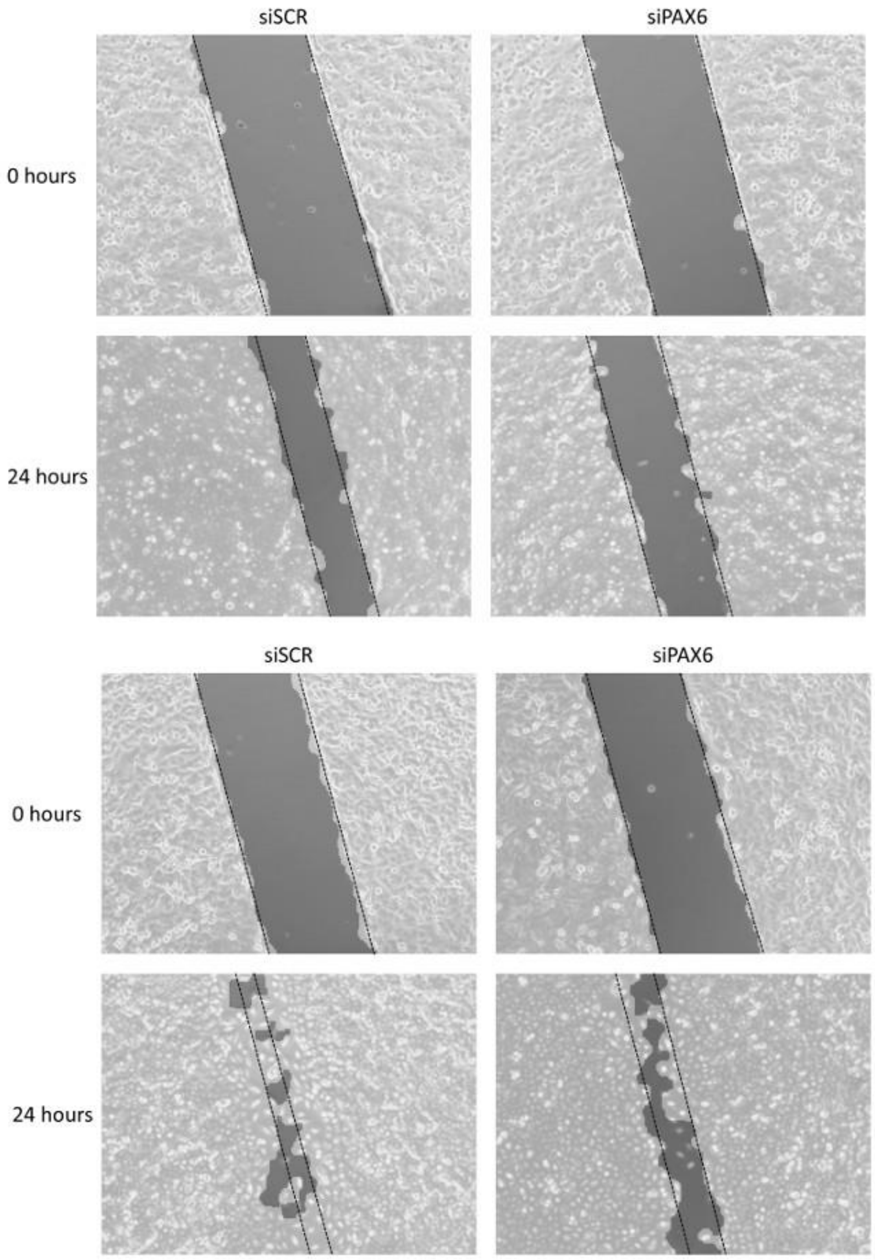

Supplement: Supplementary file 1 — Supplementary figures [file 41598_2018_23417_MOESM1_ESM.pdf]
